# The Effects of Nitrogen Alloying on the Microstructure and Properties of Cu-Bearing Antimicrobial Stainless Steel

**DOI:** 10.3390/ma18010026

**Published:** 2024-12-25

**Authors:** Yuguo Tu, Wei Peng, Liujie Chen, Xueshan Xiao

**Affiliations:** 1 School of Materials Science and Engineering, Shanghai University, Shanghai 200444, China; tuyuguo@steeljs.com (Y.T.); chenliujie@163.com (L.C.); 2 Jiangsu Shanyuan Group Co., Ltd., Taizhou 225722, China

**Keywords:** nitrogen alloyed austenitic stainless steel, Cu-rich nanoprecipitation, antibacterial, mechanical properties, direct-drinking water pipe

## Abstract

In this study, a novel Cu-bearing 304 stainless steel doped with 4.0 wt.% Cu (304-Cu SS) was developed, and the effects of nitrogen microalloying (304N-Cu SS) and heat treatment on mechanical, antibacterial, and corrosion properties were investigated. It was found that when aging at 700 °C, the Vickers hardness and strength of the 304N-Cu SS first significantly increased with increasing aging time up to 4 h and then slowly decreased with further increase in aging time. The best combination of strength and ductility, namely, a yield strength of 319 MPa, ultimate tensile strength of 657 MPa, and elongation to fracture of 47.0%, was achieved in the 304N-Cu SS after aging at 700 °C for 6 h. Moreover, the antibacterial and corrosion rates of the newly developed 304N-Cu SS reached 99.67% and 0.0032 g·m^−2^h^−1^, surpassing those of 304-Cu SS by 0.38% and 9.4%, respectively. These enhancements in the mechanical, antibacterial, and corrosion properties were attributed to the precipitation of high-density nanoscale Cu-rich precipitates during aging. Our results demonstrate that nitrogen microalloying is an effective metallurgical method for the future development of new antibacterial austenitic stainless steels with simultaneously enhanced mechanical, antibacterial, and corrosion properties for direct drinking water distribution systems.

## 1. Introduction

Human health has always been a major concern, especially regarding the quality of drinking water. However, increasing water contamination and health risks posed by pathogenic bacteria in tap water have raised public concern. It has been reported that diarrheal diseases caused by inadequate sanitation and hygiene result in 4000 to 6000 deaths per day globally, particularly among children [1]. For the safety of drinking water in daily life, antibacterial drinking water pipes are desirable. Therefore, there is an urgent need to develop effective antibacterial materials for use in tap water. Copper (Cu) is a common antibacterial element effective against many microbes, such as bacteria, fungi, and viruses [2]. In recent years, Cu-bearing antibacterial austenitic stainless steels have attracted significant attention as desirable structural and functional integrated materials [3]. These novel materials are broadly applied in many fields, including medical facilities, the food industry, household appliances, and kitchenware, owing to their excellent combination of mechanical performance, corrosion resistance, and antibacterial properties [4]. The antibacterial function of antibacterial stainless steels depends on the Cu content and appropriate aging treatment, which can precipitate nanoscale Cu-rich phases in the austenitic matrix [5,6]. It is widely recognized that dissolved Cu ions released from Cu-rich phases on the surface of a steel matrix play a crucial role in the antibacterial ability of Cu-bearing stainless steel [7,8,9]. For example, Zhang et al. indicated that a large number of Cu ions dissolved from an AISI 304 alloy surface disrupted the bacterial cell membrane, resulting in bacterial death [10]. Moreover, Li et al. concluded that the antibacterial rate of stainless steel gradually increases to a maximum with prolonged contact with bacteria, owing to the gradual increase in Cu ions dissolved from the steel matrix [11].

However, as an alloying element, Cu negatively affects the localized corrosion resistance of austenitic stainless steel in chloride environments, especially the pitting corrosion resistance, which limits its potential use in many areas [12,13]. Nitrogen (N) was intentionally introduced into the alloy to improve the corrosion resistance of austenitic stainless steel. Nitrogen is a strong austenite stabilizer that has a beneficial effect on austenitic stainless steel [14]. Furthermore, nitrogen can increase the strength and hardness without decreasing the plasticity and can effectively enhance the pitting corrosion resistance [15,16].

Most previous studies have focused on 304 Cu-bearing antibacterial austenitic stainless steel [17,18,19,20], with few addressing nitrogen-alloyed 304 Cu-bearing stainless steel. Consequently, this study investigates the Cu-rich phase precipitation behavior in a new copper- and nitrogen-alloyed austenitic stainless steel after the aging process to optimize the combination of mechanical and antibacterial properties. This study provides a scientific basis for the practical application of this material in direct drinking-water pipe systems. This could also be significant for further research on other types of antibacterial stainless steels.

## 2. Materials and Methods

### 2.1. Materials Preparation

A nitrogen-alloyed Cu-bearing 304 stainless steel material with a nominal chemical composition of Fe-Cr19-Ni10-Cu4.0-N0.2 (wt.%) (labelled as 304N-Cu SS) was prepared in a vacuum induction melting furnace under the protection of an argon atmosphere. It is noteworthy that the nitrogen alloying mainly used Cr2N alloy as raw material. For comparison, a nitrogen-free Cu-bearing 304 stainless steel material with a composition of Fe-Cr19-Ni10-Cu4.0 (wt.%) (labelled as 304-Cu SS), provided by Jiangsu Shanyuan Group Co., Ltd., Taizhou, China, was also investigated. The composition of the alloy was measured using direct reading spectroscopy, and N was measured using an oxygen/nitrogen/hydrogen analyzer (ONH-2000, ELTRA, Neuss, Germany). The 25 kg cast ingot was hot forged into bars with a thickness of 40 mm and a width of 100 mm. After soaking at 1050 °C for 1 h, the bars were hot rolled through five passes into plates with a thickness of 5 mm. Subsequently, the hot-rolled plates were cold rolled into sheets with a thickness of approximately 1.2 mm, and the deformation of cold rolling reached 76%. The rolled sheets were solution-treated at 1050 °C for 30 min, followed by water quenching. They were then aged isothermally at 700 °C for 4, 6, or 8 h, followed by air cooling to facilitate the precipitation of Cu-rich nanoparticles in the steel matrix for antibacterial functionality. Before microstructural characterization and property measurements, all the samples were ground with SiC sandpaper from 400# to 2000#, and then mechanically polished with diamond paste.

### 2.2. Microstructure Characterization

Microstructural observations of different heat-treated 304N-Cu SS samples were performed using a KEYENCE VHX-100 (KEYENCE, Osaka, Japan) optical microscope (OM), after they were etched in a 10% oxalic acid solution at 20 V for 30 s. More detailed microstructural information of the 304N-Cu SS after aging at 700 °C for 6 h was obtained using a JEM-2100F (JEOL, Tokyo, Japan) transmissionelectron microscope (TEM) at an operating voltage of 200 kV. The TEM sample was ground into a slice with a thickness of approximately 100 μm and cut into thin foils with a diameter of 3 mm. Subsequently, the thin foils for TEM observation were mechanically ground to a thickness of approximately 55 μm and then thinned using an MTP-1A type twin jet electrolytic polishing system in a 5 vol.% HClO_4_ ethanol electrolyte solution at −30 °C and 35 V. Atomic-scale chemical analysis of 304N-Cu SS after aging at 700 °C for 6 h was performed using a LEAP 4000X HR three-dimensional (Imago Scientific Instruments, Madison, WI, USA) atom probe (3DAP). The 3DAP starting samples with a cross-section of 0.5 × 0.5 mm^2^ and a length of 15 mm were cut from the aged steel and were electropolished to sharp needles using a standard two-step electropolishing technique. Atom probe tomography (APT) was performed in an analyzed volume of 68 × 69 × 348 nm^3^ at a base temperature of 50 K, pulse energy of 60 PJ, and pulse repetition rate of 200 kHz. Data reconstruction and statistical evaluation were conducted using the IVASTM 3.6.14 software package.

### 2.3. Mechanical Performance Testing

The microhardness of the 304N-Cu SS and 304-Cu SS samples was measured using an HXD-1000TMC/LCD (Shangguang, Shanghai, China) type digital Vickers hardness tester with a load of 200 g and testing time of 15 s. The microhardness value was derived from an average of 8 different points on each sample. Tensile tests were conducted using a universal testing machine at room temperature at a strain rate of 1 × 10^−3^ s^−1^. The dog-bone-shaped specimens with a cross section of 1.2 mm × 25 mm and a gage length of 50 mm were cut by electrical discharge machining in accordance with International Standards ISO 6892-1:2009 [21]. Three parallel specimens were tested, and the average test results were obtained

### 2.4. Antibacterial Testing

The plate counting method was used to evaluate the antibacterial properties of the two steel samples aged at 700 °C for different durations by quantifying the number of live bacteria. A solution-treated steel sample was used as control. All specimens with dimensions of 20 mm × 20 mm × 1 mm were mechanically polished with 2000 grit SiC paper. The antibacterial tests were performed according to GB/T standard 10124-1988 [22]. The test media and samples were sterilized by autoclaving at 121 °C for 20 min before testing. *Escherichia coli* (*E. coli*) ATCC25922 was used as the testing bacterium and cultured in a standard medium containing 5.0 g/L flesh extract, 5.0 g/L NaCl, 10.0 g/L peptone, 20.0 g/L agar, and 1000 mL distilled water, with the pH value adjusted to 7.2 ± 0.1. A bacterial solution (0.5 mL) with a concentration of 10^5^ CFU/mL was dropped onto the test samples. The specimens were covered with an aseptic polyethylene film to ensure that the bacterial solution was in close contact with the surfaces of the samples. The cells were then incubated at 37 °C for 24 h under 90% humidity. After incubation, the bacterial solution was diluted to 10^3^ CFU/mL, and the diluted bacterial solution (0.1 mL) was added to nutrient agar plates, which were incubated at 37 °C and 90% humidity for another 24 h to count the number of bacterial colonies.

The antibacterial rate was calculated using the following Equation [23]:

(1) $$K=\frac{(A-B)}{A}\times 100\%$$

where *K* is the antibacterial rate, *A* is the number of bacterial colonies of the solution-treated 304N-Cu SS, and *B* is the number of bacterial colonies of the 304N-Cu SS with varying aging treatments. Each test was repeated thrice, and the average value was taken.

### 2.5. Corrosion Resistance

For the full-immersion corrosion test, 304N-Cu-SS and 304-Cu-SS specimens aged for 6 h at 700 °C were selected. Samples with dimensions of 50 mm × 25 mm × 1 mm were cut by electrical discharge machining and then ground with sandpaper from 150# to 2000#. Subsequently, the samples were cleaned with acetone to remove oil, followed by ultrasonic cleaning in anhydrous ethanol for 30 min, removal with forceps and blown dry, and finally dried in a drying oven. After drying, the samples were weighed, and the initial mass of each sample was recorded. The samples were then placed in a water bath at constant temperature for 120, 240, 360, and 480 h.

As stainless steel is primarily utilized in direct drinking water pipes, it is critical to simulate its corrosion resistance in the presence of chloride ions. A 3.5% NaCl solution (PH 7.5) at 30 °C was used to simulate the test environment according to the GB/T Standard 10124-1988. The solution volume to the surface area ratio was maintained at not less than 20 mL/cm^2^. The test period was 120 h, and the solution was changed every period, ensuring a consistent solution volume each time. Triplicate samples were used for each group of tests, and the test temperature was maintained at 30 ± 1 °C. After removal from the solution, the tested samples were first rinsed with water, and the corrosion products were removed by ultrasonic cleaning according to the GB/T Standard 10124-1988, and the samples were then blow-dried and weighed using a BT 125D electronic balance weighing system with an accuracy of 0.01 mg.

(2) $$v_{cor}=(m_{1}-m_{2})\cdot S^{-1}\cdot t^{-1}$$

where $v_{cor}$ is the corrosion rate, $m_{1}$ is the mass of the sample before the test, $m_{2}$ is the mass after the test, S is the surface area of the sample, and t is the testing time.

The polarization curves of the materials were acquired using an electrochemical workstation at a temperature of 25 °C in a 3.5% NaCl solution with a scan rate of 1 mV/s and a scanning range from −300 mV to 700 mV. A saturated KCl-filled Ag/AgCl electrode was used as the reference electrode, and a platinum electrode was used as the auxiliary electrode, as outlined in reference [24]. Three analyses were conducted for each material to ensure reproducibility of the results.

## 3. Results and Discussion

### 3.1. Mechanical Properties

**Figure 1:**
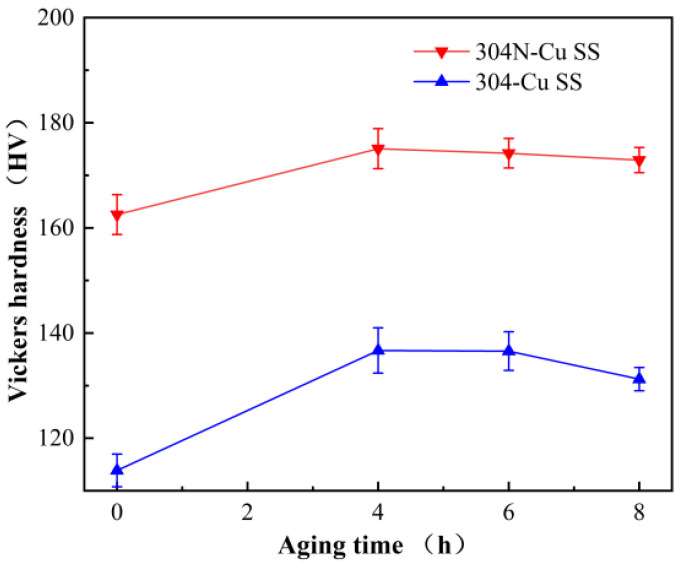
Microhardness of 304N-Cu SS and 304-Cu SS with aging time.

The microhardness variations of the 304N-Cu SS and 304-Cu SS after solution and aged treatments at 700 °C for 4, 6, and 8 h are shown in Figure 1. As N is an austenite-forming element with an interstitial solution strengthening effect, it improves the strength of steel, and consequently, nitrogen-containing 304N-Cu SS exhibits significantly higher microhardness and tensile strength than 304-Cu SS. The microhardness of solution-treated 304N-Cu SS was approximately 162 HV. After aging for 4 h, the microhardness increased significantly to a peak value of 175 HV, indicating the occurrence of precipitation hardening. The microhardness then exhibited a slight decrease with increasing aging time from 4 to 8 h. Defects such as vacancies, dislocations, grain boundaries, and twin boundaries, which are present in solution-treated samples, not only provide favorable nucleation sites for Cu precipitates but also promote the diffusion of oversaturated Cu atoms [25]. The rapid formation of Cu-rich precipitates in the steel during the early aging stage resulted in a rapid increase in both the radius and volume fraction of the Cu-rich precipitates. Consequently, the microhardness of the 304N-Cu SS and 304-Cu SS was markedly enhanced during the aging period of 0 to 4 h. With an increase in aging time, the coarsening of the Cu-rich phases decelerated, and the number of Cu-rich precipitates exhibited minimal variation. Therefore, the microhardness values of the 304N-Cu SS and 304-Cu SS specimens decreased slightly [26].

**Figure 2:**
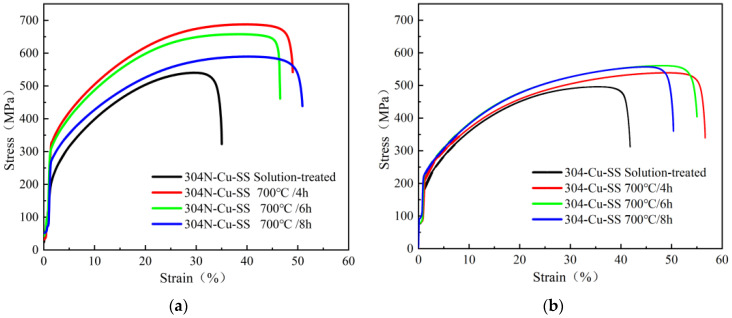
Room-temperature tensile engineering stress–strain curves under different heat-treatment conditions: (**a**) 304N-Cu SS; and (**b**) 304-Cu SS.

Figure 2 shows the room-temperature uniaxial tension engineering stress-strain curves of the 304N-Cu SS and 304-Cu SS specimens after solution treatment and aging at 700 °C for 4, 6, and 8 h. Table 1 lists the measured mechanical properties. Both 304N-Cu SS and 304-Cu SS exhibited increased ultimate tensile strength (UTS) after aging; however, 304N-Cu SS was stronger than 304-Cu SS after the same aging time, which is consistent with the Vickers hardness test results (Figure 1). This demonstrates a substantial increase in strength, proving that the experimental steel could be effectively strengthened through precipitation hardening [27]. The UTS of 304N-Cu SS was 657 MPa after aging at 700 °C for 6 h, whereas the UTS of 304-Cu SS was 560 MPa after 6 h. Therefore, it can be inferred that nitrogen effectively improves the strength of Cu-bearing austenitic stainless steel. Based on thermodynamic calculations of the activity coefficient of copper in the Fe-Cr-Ni-Cu-N-C-Si-Mn alloy system and its interaction with nitrogen [15], it was found that nitrogen increases the activity coefficient of copper in the alloy, which increases the free energy difference in the copper concentration distribution between the precipitates and austenite matrix, promoting the precipitation of the Cu-rich phase. During the deformation process, these randomly distributed Cu-rich precipitates accumulated and pinned dislocations. As a result, the strength of the 304N-Cu SS effectively increased. In addition, the free nitrogen atoms in the interstitial sites increased the bond forces in the lattice, causing interstitial solid-solution strengthening. It has been demonstrated that the degree of strengthening is directly proportional to the quantity of nitrogen present [16]. Hence, although the increase of strength resulted in a decrease of elongation, the degree of elongation reduction is not much, which can meet the needs of general manufacturing.

### 3.2. Microstructure Observation

**Figure 3:**
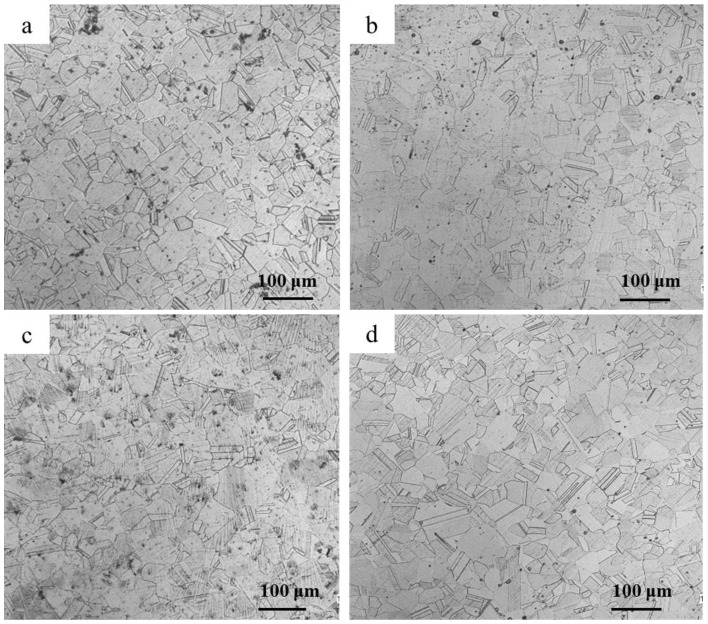
OM images of the microstructures of 304N-Cu SS after different heat treatments: (**a**) solution-treated and (**b**–**d**) aging at 700 °C for 4, 6, and 8 h, respectively.

Figure 3 shows the microstructure of 304N-Cu SS after solution treatment and aging at 700 °C for 4, 6, and 8 h. The microstructures of the specimens in solution with different aging times consisted entirely of equiaxed polygonal austenite grains with annealing twins. The twin formation is characteristic of austenitic steels and other face-centered cubic (FCC) metals and alloys with low stacking fault energies when subjected to solution treatment after plastic deformation [28]. Comparing Figure 3a–d, it can be seen that the precipitation of Cu-rich nanoparticles had almost no effect on the grain size of the austenitic matrix, but the number of twins decreased with longer annealing times.

**Figure 4:**
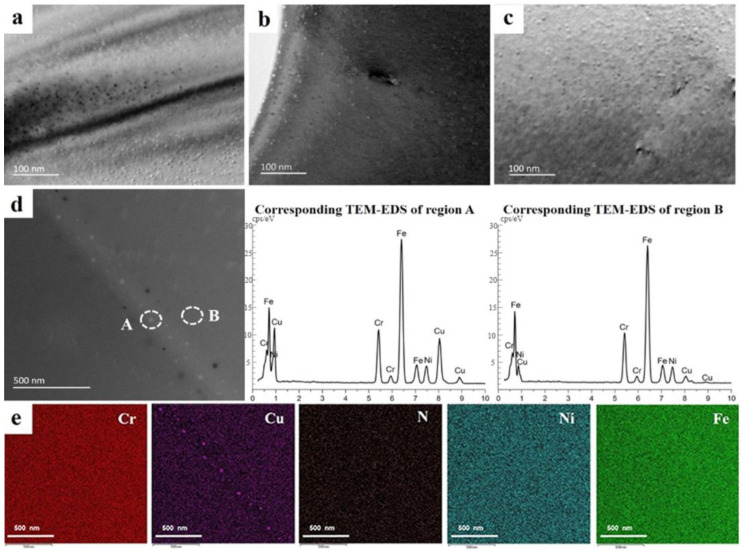
TEM micrographs of the experimental steel after aging at 700 °C for (**a**) 4 h, (**b**) 6 h, and (**c**) 8 h. (**d**) Cu-rich precipitation at the grain boundary after aging for 6 h, with corresponding EDS analysis of the selected areas of the Cu-rich precipitate (A) and matrix (B). (**e**) TEM-EDS mapping analysis of the area in (**d**).

TEM micrographs of the 304N-Cu SS samples after aging at 700 °C for 4, 6, and 8 h are shown in Figure 4a–c. The high-density nanoprecipitates were uniformly distributed in the matrix. As aging time increased, the precipitates grew slightly. When the aging time was increased to 8 h, the number of precipitates slightly decreased. It can be observed that a large number of spherical nanoprecipitates are distributed within the steel matrix after aging at 700 °C for 6 h, with diameters ranging from to 3–5 nm. Figure 4d shows a high-angle annular dark-field (HAADF) image of the nanoprecipitates at the grain boundaries after aging at 700 °C for 6 h, along with energy dispersive spectroscopy (EDS) analyses of the precipitates (A) and steel matrix (B), revealing that the precipitates exhibit a higher Cu content than the steel matrix. These results indicate that the precipitates were in the Cu-rich phase. The TEM-EDS mapping analysis of the entire area in Figure 4 is shown in Figure 4e, indicating that the precipitates at the grain boundaries were rich in Cu. Simultaneously, other elements such as Cr, N, Ni, and Fe were distributed uniformly throughout the matrix. The Cu-rich precipitates at the grain boundaries were larger than those within the grain interiors. This is due to the presence of many defects at the grain boundaries, providing more rapid diffusion channels for Cu atoms, thus leading to the segregation of atoms. Therefore, the nucleation and growth rates of the Cu-rich precipitates are promoted during aging.

**Figure 5:**
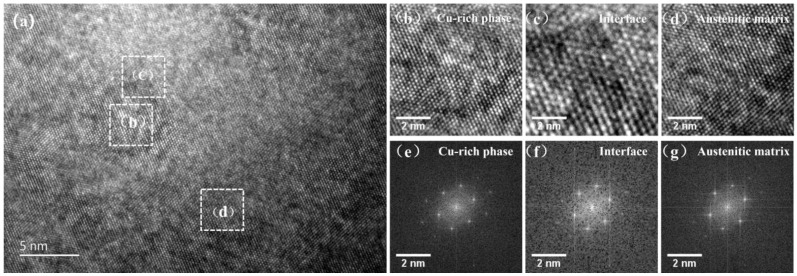
(**a**) HRTEM image of Cu-rich precipitates in 304N-Cu SS after aging at 700 °C for 6 h. Enlarged images of (**b**) Cu-rich precipitate, (**c**) interface, and (**d**) austenitic matrix. The corresponding FTT images of the Cu-rich phase, interface, and matrix are shown in (**e**–**g**).

In addition, high-resolution transmission electron microscopy (HRTEM) was used to further investigate the crystal structure of the Cu-rich precipitates in the 304N-Cu SS sample after aging at 700 °C for 6 h, as shown in Figure 5a. Figure 5e–g exhibits the corresponding fast Fourier transform (FFT) images of the Cu-rich precipitates, precipitate/matrix interface, and the matrix, respectively. The HRTEM results revealed that the Cu-rich precipitates were completely coherent with the matrix. The reasons for this are as follows. Firstly, unlike ferritic steels, there is no phase transformation accompanying the growth of Cu-rich precipitates in austenitic steels, which directly forms a stable FCC structure [29,30]. Secondly, the atomic radius of Fe (0.124 nm) is almost equal to that of Cu (0.128 nm), resulting in a rather small mismatch between the FCC Cu-rich phase and matrix [31]. Combined with the TEM-EDS analysis and the structure of the Cu-rich phase, the precipitation was thus defined as an *ɛ*-phase structure.

### 3.3. APT Characterization of Cu-Rich Precipitates

**Figure 6:**
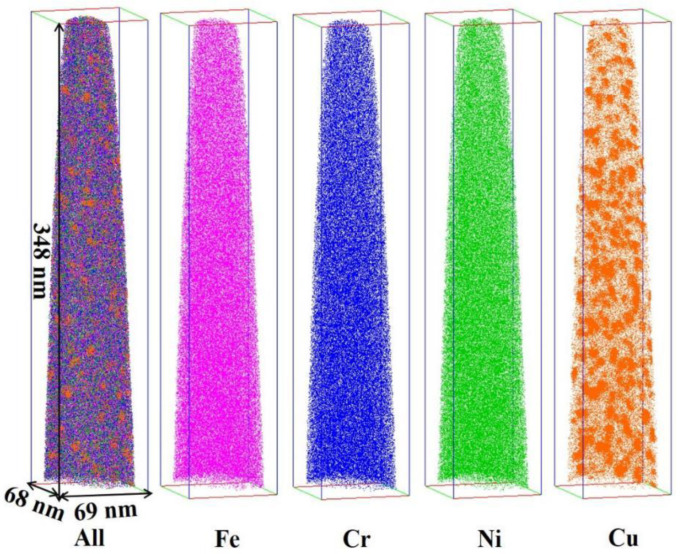
3D atom-by-atom tomographic reconstruction of 304N-Cu SS after aging at 700 °C for 6 h. The volume is 68 nm × 69 nm × 348 nm.

APT analysis was used to determine the chemical composition and size of the Cu-rich precipitates. Figure 6 shows the 3D atom-by-atom tomographic reconstruction of 304N-Cu SS after aging at 700 °C for 6 h. Only Cu was significantly segregated, while other elements such as Fe, Cr, and Ni were distributed homogeneously, consistent with the TEM results.

The distribution of Cu-rich precipitates in 304N-Cu stainless steel in the 6 h aging state was first determined by the maximum separation envelope method (MSEM) [32]. The parameter set of separation distance (SP) and minimum number (MN) was set as 0.5 nm and 20 nm, respectively. The equivalent radius Rp of the *ɛ*-Cu phase in the 6-h aging state was calculated to be 3.4 nm, and the number density Nv was 5.42 × 10^23^ m^−3^. As shown in Equation (3), the equivalent spacing of the Cu-rich precipitates, *L*, can be calculated [33]. The equivalent spacing of the Cu phase L was calculated to be 20.2 nm.

(3) $$L=0.866\times {\left(R_{p}N_{v}\right)}^{-\frac{1}{2}}$$

**Figure 7:**
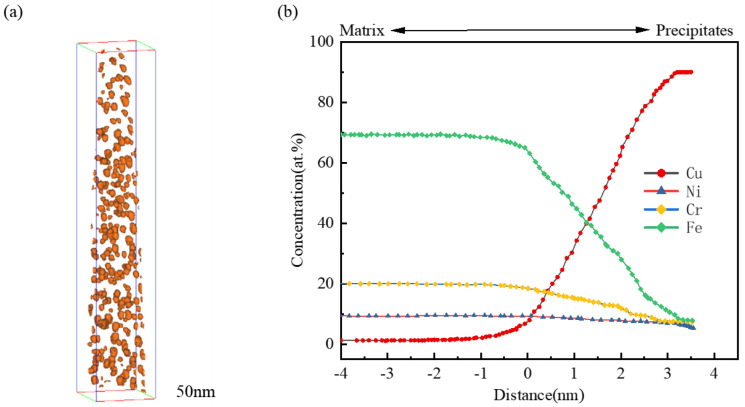
Three-dimensional spatial distribution of Cu atoms in 304N-Cu SS after aging at 700 °C for 6 h. (**a**) Cu = 10% isoconcentration surface of the precipitate. (**b**) Concentration distribution across the matrix–precipitate interface.

To further analyze the enrichment of Cu in 304N-Cu SS, the Cu = 10% isoconcentration surface of the precipitates in 304N-Cu SS after 6 h of aging was selected, as shown in Figure 7a. Figure 7b depicts the elemental distribution between the matrix and precipitate, revealing that the Cu content in the matrix of 304N-Cu SS was 1.1.%. After 6 h of aging, the Cu content at the center of the Cu-rich phase increased dramatically to 90.1%, significantly exceeding that of the matrix. In contrast, the concentrations of Fe, Cr, and Ni were noticeably lower in the Cu-rich precipitates than in the matrix.

### 3.4. Antibacterial Properties

**Figure 8:**
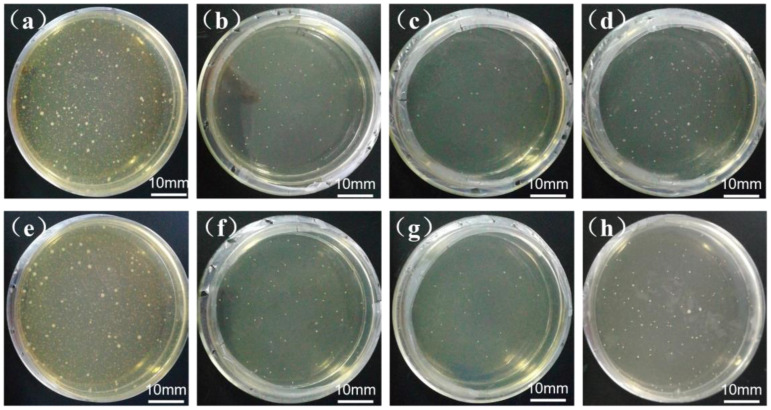
Photographs of bacterial colonies of the experimental steels in *E. coli* suspension: (**a**) control sample 1, and (**b**–**d**) 304N-Cu SS aging at 700 °C for 4, 6, and 8 h, respectively; (**e**) control sample 2, and (**f**–**h**) 304-Cu SS aging at 700 °C for 4 h, 6 h, and 8 h, respectively.

The plate machine counting method was used to assess the antibacterial effectiveness of the experimental steels against *Escherichia coli*. Figure 8 shows the antibacterial effectiveness of 304N-Cu SS and 304-Cu SS after aging at 700 °C for 4, 6, and 8 h. As illustrated in Figure 8a,e, the control samples were both solid-solution treated, which exhibited a substantial number of colonies in the petri dishes after 24 h of incubation, indicating that there were little apparent antibacterial properties. In contrast, as shown in Figure 8b–d,f–h, the number of colonies in the Petri dishes of the two tested steels after aging was significantly reduced, demonstrating that bacterial growth was effectively suppressed in the copper-containing stainless steels. Table 2 lists the measured antibacterial rates of the experimental steels against *E. coli*. The bacterial counts on the 304N-Cu SS and 304-Cu SS standard plates that had been solution treated were nearly equal to those on the control plate, which showed insignificant antimicrobial activity. According to Equation (1), the 304N-Cu SS after aging at 700 °C for 4, 6, and 8 h exhibited antibacterial rates of 98.71, 99.67, and 97.51%, respectively, whereas those of the 304-Cu SS specimens aged at 700 °C for 4, 6, and 8 h, is 98.27%, 99.29%, and 97.20%, respectively. After 6 h of aging, both test steels achieved peak antibacterial activity. However, under identical aging conditions, 304N-Cu SS exhibited slightly better antibacterial properties than 304-Cu SS. In terms of the antibacterial process, it has been established that the Cu^2+^ produced from the Cu-rich precipitates penetrates the bacteria and kills them [34]. The superior antibacterial property depends on the Cu^2+^ content, and the antibacterial action is only activated when the concentration of released Cu^2+^ exceeds a critical value [35,36]. The experimental steel responded optimally to aging at 700 °C for 6 h, as the increased number density of nanoscale Cu-rich precipitates correlated with higher strength and antibacterial rates. According to the study [15], which calculated the interaction activity coefficient between nitrogen and alloyed copper in a multicomponent alloy containing high content chromium and nickel, nitrogen in the alloy increases the activity coefficient of copper, thereby promoting the precipitation of Cu-rich precipitates.

As a result, the 304N-Cu SS and 304-Cu SS manifested similar antibacterial activity, which can be rightfully attributed to their Cu contents. Additionally, the nitrogen-containing 304N-Cu SS contains more *ɛ*-Cu precipitates, which significantly improved mechanical properties and thus its practical applications.

### 3.5. Corrosion Rate Determination

**Figure 9:**
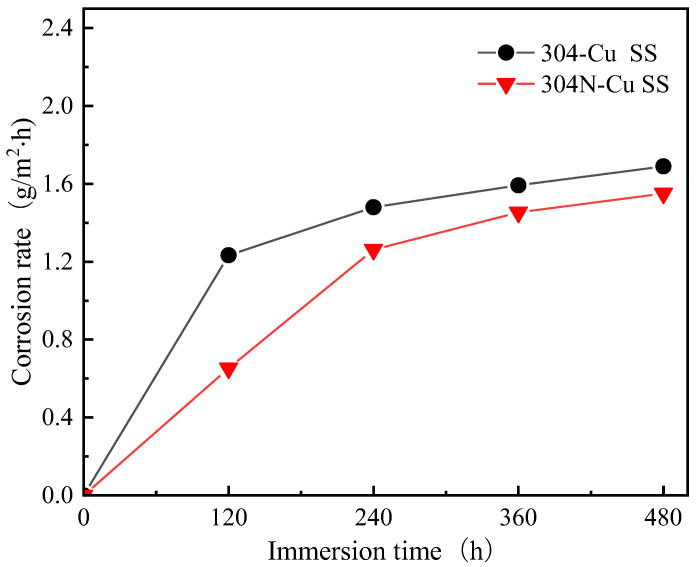
The corrosion rate curves of aged 304-Cu SS and 304N-Cu SS immersing in 3.5 wt.% NaCl at 35 °C for 480 h.

**Figure 10:**
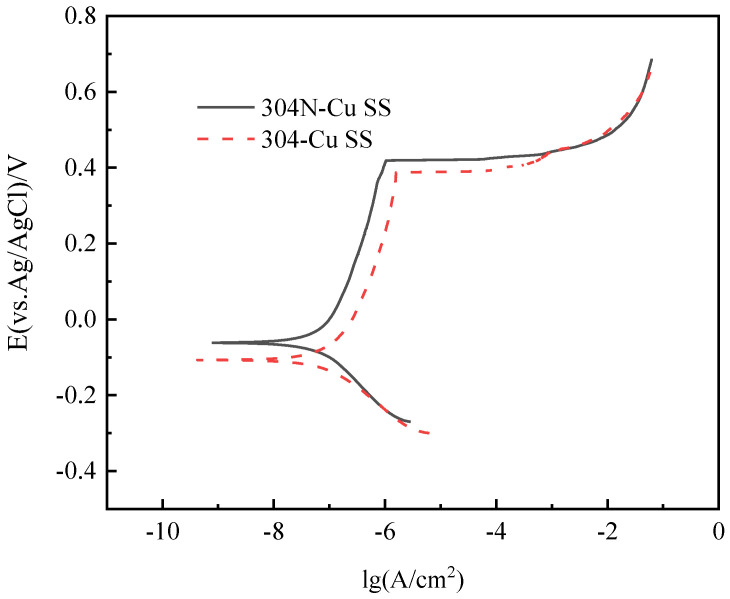
Anodic polarization curves of 304N-Cu SS and 304-Cu SS at 25 °C in a 3.5% NaCl solution.

The corrosion rate curves of 700 °C/6 h-aged 304-Cu SS and 304N-Cu SS immersing in 3.5 wt.% NaCl at 35 °C for 480 h are shown in Figure 9. The corrosion rates of 304N-Cu-SS is 0.0032 g·m^−2^h^−1^, while that of 304-Cu-SS is 0.0035 g·m^−2^h^−1^. This indicates that 304N-Cu SS exhibited better corrosion resistance than 304-Cu SS. Figure 10 presents the polarization curves of 304N-Cu SS and 304-Cu SS in 3.5% sodium chloride solution at 25 °C. The detailed electrochemical parameters obtained using Tafel extrapolation are listed in Table 3. The data presented in Table 3 demonstrates that the corrosion potential (*E*_corr_: described as the lowest potential at which the reaction rate of the cathodic process equals that of the anodic process) of 304N-Cu SS is −0.073 V SCE, which is slightly higher than that of 304-Cu SS steel (−0.113 V SCE). However, the corrosion current (*i*_corr_: acquired by the intersection of the Tafel part of the cathodic branch) was larger in 304-Cu SS steel than in 304N-Cu SS.

The pitting potential (*E_b_*) is frequently employed to evaluate the resistance of a material to localized corrosion (chiefly pitting), which is regarded as the predominant form of corrosion in passive alloys and defined as the potential at which the anodic current density is 100 μA/cm^2^. An elevated *E_b_* value is indicative of an enhanced resistance to pitting. The *E_b_* of 304-Cu SS steel without nitrogen was lower, and the *i_corr_* of the nitrogen-alloyed 304N-Cu SS was smaller. The data indicates that 304 stainless steels alloyed with nitrogen exhibit enhanced corrosion resistance, which is in accordance with the results presented in Figure 9. It is evident that the corrosion rate of 304N-Cu SS with a higher nitrogen content is lower, thereby rendering it a more suitable material for use in direct drinking water pipes. It has been documented that the *E_b_* and *i_corr_* values of aged 304-Cu SS measured in a 3.5% NaCl solution was 0.171 V SCE and 0.1 × 10^−2^ A/m^2^, respectively, which are comparable to the data obtained in this study [37].

**Figure 11:**
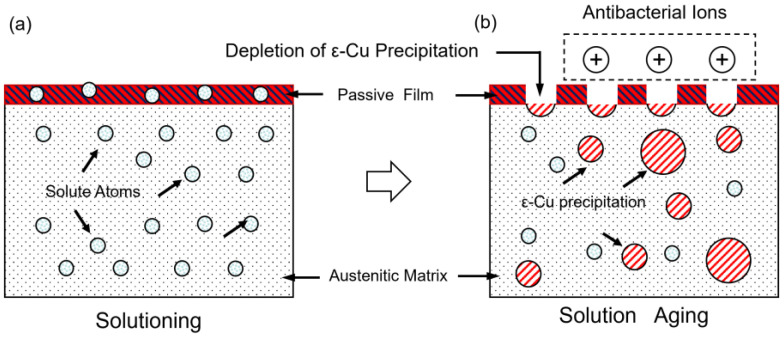
Schematic illustration of discontinuous passivation film and *ɛ*-Cu depletion. (**a**) Solution state. (**b**) Aged state.

As shown in Figure 11, it has been demonstrated that the precipitation of the *ɛ*-Cu phase after the surface aging treatment has a deleterious impact on the corrosion resistance of stainless steel. The dissolution of copper during the solution treatment of stainless steel does not compromise the stability of the passivated oxide film. However, following the aging treatment, the *ɛ*-Cu phase precipitates on the passivation film, thereby reducing the corrosion resistance of stainless steel in NaCl solution [38]. This phenomenon is attributable to the galvanic corrosion process that occurs between *ɛ*-Cu precipitates on the passivation film. In NaCl solution, the corrosion potential of *ɛ*-Cu exceeds that of austenitic stainless steel [39]. The presence of active Cu precipitates on the passivation film renders the Cu phases unprotected by the Cr_2_O_3_ passivation film, which renders them susceptible to corrosion [40]. Hence, it can be inferred that the *ɛ*-Cu phase precipitates represent a ‘weak point’ in the passivation film, resulting in the destruction of continuity. As illustrated in Figure 11b, the formation of discontinuous passivation films reduced the ability of stainless steel to resist pitting corrosion. At the same time, the activated *ɛ*-Cu is released as charged Cu^2+^ to form antimicrobial ions.

In addition, Zhang et al. [41] found that nitrogen on the surface of alloy 304 counteracted the detrimental effects of copper precipitates on the corrosion performance by performing microstructural and compositional studies using AFM and AES. The addition of nitrogen to 304N-Cu SS can potentially improve the corrosion resistance of the material. This is mainly because the nitrogen enrichment on the surface of the alloy plays a role in improving the passivation ability of the steel and the stability of the passivation film, and nitrogen inhibits the decrease in the pH value of the micro-zone solution, which is favorable for the passivation of the steel. This is in agreement with the results of the present study, which may be responsible for the improved corrosion resistance after the addition of nitrogen to austenitic antimicrobial stainless steels.

## 4. Conclusions

This study demonstrated that the hardness of 304N-Cu SS increased rapidly at the early stage of aging because of the fast precipitation of Cu-rich precipitates and then tended to stabilize with a further increase in aging time from 4 to 8 h. The strength of the 304N-Cu SS increased, whereas the elongation decreased with aging time. The ultimate tensile strength of 304N-Cu SS after solution and aging was significantly higher than that of 304-Cu SS under the same heat treatment, indicating that nitrogen effectively improved the strength of the Cu-bearing austenite stainless steel. Additionally, the 304N-Cu SS exhibited better corrosion resistance than the 304-Cu SS. Overall, 304N-Cu SS exhibits an excellent combination of mechanical properties, corrosion resistance, and antibacterial properties, which surpasses 304-Cu SS to become a promising candidate for direct drinking water distribution networks.

## Figures and Tables

**Table 1:** The mechanical properties of 304N-Cu SS and 304-Cu SS under different heat treatment conditions.

Steel Type	Heat Treatment	UTS (MPa)	YS (MPa)	EL (%)	RA (%)
304N-Cu SS	Solution-treated	540 ± 8	229 ± 7	35.4 ± 3.2	74.7 ± 4.4
	700 °C/4 h	687 ± 5	346 ± 12	48.7 ± 1.6	73.9 ± 3.1
	700 °C/6 h	657 ± 9	328 ± 6	46.5 ± 2.7	76.7 ± 4.3
	700 °C/8 h	589 ± 10	288 ± 9	50.9 ± 3.0	73.3 ± 3.8
304-Cu SS	Solution-treated	496 ± 7	218 ± 5	41.8 ± 1.8	74.5 ± 2.9
	700 °C/4 h	538 ± 4	249 ± 2	56.7 ± 4.1	76.4 ± 4.5
	700 °C/6 h	560 ± 11	250 ± 9	55.0 ± 3.7	75.5 ± 3.3
	700 °C/8 h	557 ± 6	248 ± 3	50.4 ± 1.6	74.4 ± 2.8

**Table 2:** Antibacterial rates of aged 304N-Cu SS and 304-Cu SS against *E. coli*.

Steel Type	Heat Treatment	Cultivated Bacterial Count (CFU/mL)	Cultivated Bacterial Count After 24 h (CFU/mL)	Antibacterial Rate (%)
304N-Cu SS	700 °C/4 h	4.5 × 10^3^	58	98.71
	700 °C/6 h		15	99.67
	700 °C/8 h		112	97.51
304-Cu SS	700 °C/4 h	4.5 × 10^3^	78	98.27
	700 °C/6 h		32	99.29
	700 °C/8 h		126	97.20

**Table 3:** Electrochemical parameters of 304N-Cu SS and 304-Cu SS in 3.5% NaCl solution.

Steel Type	*E*_corr_ (V)	*E*_b_ (V)	*i*_corr_ (A/cm^2^)
304N-Cu SS	−0.073	0.426	0.785 × 10^−7^
304-Cu SS	−0.113	0.401	1.023 × 10^−7^

*E_corr_* denotes corrosion potential, *i_corr_* corrosion current, *E_b_* pitting potential.

## Data Availability

The original contributions presented in this study are included in the article. Further inquiries can be directed to the corresponding authors.
